# Roles of the Linker Region of RNA Helicase A in HIV-1 RNA Metabolism

**DOI:** 10.1371/journal.pone.0078596

**Published:** 2013-11-06

**Authors:** Li Xing, Meijuan Niu, Xia Zhao, Lawrence Kleiman

**Affiliations:** 1 Lady Davis Institute for Medical Research and McGill AIDS Centre, Jewish General Hospital, Montreal, Quebec, Canada; 2 Department of Medicine, McGill University, Montreal, Quebec, Canada; Institut National de la Santé et de la Recherche Médicale, France

## Abstract

RNA helicase A (RHA) promotes multiple steps in HIV-1 production including transcription and translation of viral RNA, annealing of primer tRNA^Lys3^ to viral RNA, and elevating the ratio of unspliced to spliced viral RNA. At its amino terminus are two double-stranded RNA binding domains (dsRBDs) that are essential for RHA-viral RNA interaction. Linking the dsRBDs to the core helicase domain is a linker region containing 6 predicted helices. Working *in vitro* with purified mutant RHAs containing deletions of individual helices reveals that this region may regulate the enzyme's helicase activity, since deletion of helix 2 or 3 reduces the rate of unwinding RNA by RHA. The biological significance of this finding was then examined during HIV-1 production. Deletions in the linker region do not significantly affect either RHA-HIV-1 RNA interaction *in vivo* or the incorporation of mutant RHAs into progeny virions. While the partial reduction in helicase activity of mutant RHA containing a deletion of helices 2 or 3 does not reduce the ability of RHA to stimulate viral RNA synthesis, the promotion of tRNA^Lys3^ annealing to viral RNA is blocked. In contrast, deletion of helices 4 or 5 does not affect the ability of RHA to promote tRNA^Lys3^ annealing, but reduces its ability to stimulate viral RNA synthesis. Additionally, RHA stimulation of viral RNA synthesis results in an increased ratio of unspliced to spliced viral RNA, and this increase is not inhibited by deletions in the linker region, nor is the pattern of splicing changed within the ∼ 4.0 kb or ∼ 1.8 kb HIV-1 RNA classes, suggesting that RHA's effect on suppressing splicing is confined mainly to the first 5′-splice donor site. Overall, the differential responses to the mutations in the linker region of RHA reveal that RHA participates in HIV-1 RNA metabolism by multiple distinct mechanisms.

## Introduction

HIV-1, like other retroviruses, does not encode its own RNA helicases, but employs cellular counterparts to facilitate its replication [1]. A number of RNA helicases have been identified to participate in HIV-1 replication including RNA helicase A (RHA) [2], DEAD box RNA helicases DDX3 [3] and DDX24 [4].

RHA, a DExH box protein, is a member of the ATP-dependent helicase superfamily 2 (SF2). It can unwind both double-stranded RNA (dsRNA) and double-stranded DNA (dsDNA) by hydrolyzing any of the four ribo- or deoxyribo- nucleotide triphosphates (NTP) [5], [6]. RHA has been found associated with a set of protein complexes related to RNA metabolism including the nuclear pore complex [7], the RNA-induced silencing complex (RISC) in the RNAi pathway [8], the Rev/Rev-response element (RRE) complex of HIV-1[9], and the spliceosome [10]. RHA plays multiple roles in HIV-1 replication, including stimulation of viral RNA synthesis [11], generation of unspliced viral RNA [2], enhancing translation [12], and promoting the annealing to viral RNA of tRNA^Lys3^, the primer for reverse transcription of HIV-1 [13], [14].

**Figure 1:**
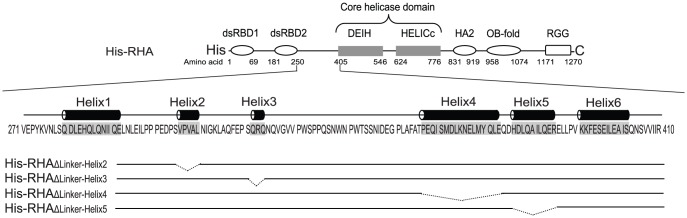
Schematic representation of the domain structure of RHA and the deletions introduced into the linker region. The proteins are tagged with 6×His at the N-terminus. Shown are the sequence and the relative positions of helices 1 to 6 in the linker region of RHA. Dotted lines represent the deleted helices.

RHA consists of 7 domains (depicted in Figure 1). A centrally located core helicase domain is composed of two RecA-like sub-domains, the DEIH sub-domain and the HELICc subdomain [15]. This core helicase domain is responsible for binding and hydrolysis of NTP, and DNA or RNA unwinding activity. The C-terminus of RHA contains a stretch of repeated arginine and glycine-glycine (RGG) residues that non-specifically bind RNA [6]. There are two conserved domains that have been identified by sequence homology between the core helicase and the RGG domains. One is the helicase-associated domain 2 (HA2) belonging to the Pfam PF04408 superfamily [16], [17]. Its function is unknown. The other is the oligonucleotide- or oligosaccha-ride-binding (OB)-fold formerly known as a domain of unknown function (DUF1605), belonging to the Pfam PF07717 superfamily [16], [17].

RHA is characterized by two double-stranded RNA binding domains (dsRBD1 and dsRBD2) at the N-terminus [18]. Both dsRBDs are essential for the interaction of RHA with HIV-1 RNA during virus replication [19]. Because dsRBDs are linked to the core helicase domain by a region of about 100 amino acids in length (named hereafter as the linker region), we have investigated by deletion mutation analysis whether this region has any influence over the helicase activity of the enzyme and upon its ability to promote various biological processes.

The linker region of RHA (Figure 1) contains 6 helices that have been predicted using the PSIPRED protein structure prediction server [20]. Deletion of each helix is shown in Figure 1, and we have studied the effect of deletions of helices 2, 3, 4, or 5 upon the ability of RHA to bind to and to unwind duplex RNA *in vitro* (we were unable to purify mutant RHA containing deletions of either helix 1 or 6). We have also examined the ability of mutant RHAs to promote some steps of HIV-1 RNA metabolism *in vivo* and have found that while helices 2 and 3 are indispensable for the promotion of tRNA^Lys3^ annealing to viral RNA, helices 4 and 5 are more critical for stimulation of viral RNA synthesis, indicating the distinct mechanisms responsible for RHA to participate in these two viral processes. Additionally, both wild-type RHA and RHA containing linker region mutations inhibit the splicing events required for the generation of singly (∼ 4.0 kb) and multiply (∼ 1.8 kb) spliced HIV-1 RNA species, resulting in a greater production of unspliced (∼ 9.2 kb) HIV-1 RNA. However, the splicing patterns within the ∼ 4.0 kb or ∼1.8 kb HIV-1 RNA classes remain unchanged, suggesting that RHA's effect on suppressing splicing is confined mainly to the first 5′-splice donor site, SD1.

## Results

### RNA binding and helicase properties of mutant RHAs

**Figure 2:**
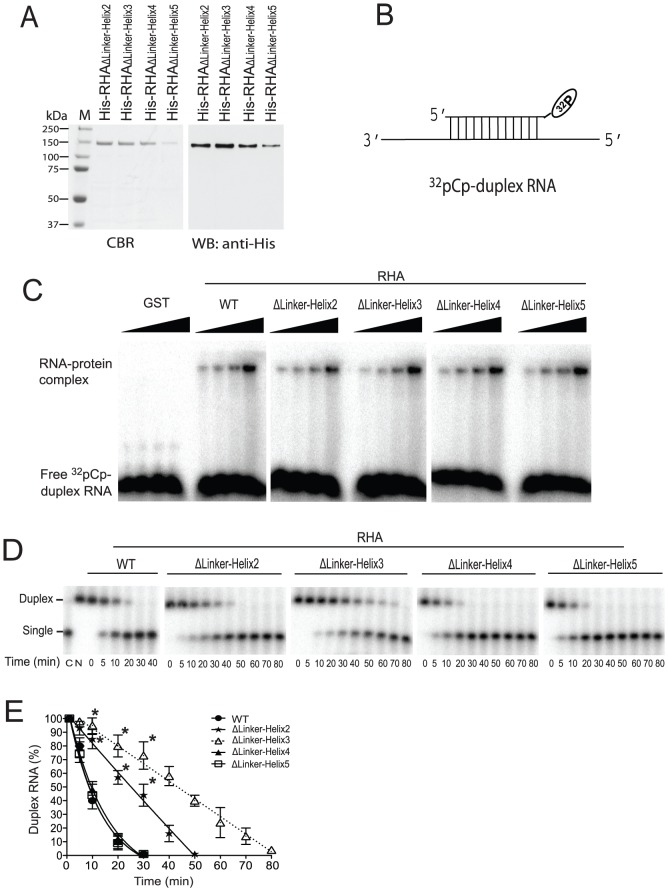
Ability of purified mutant RHAs to bind and unwind dsRNA *in vitro*. (A) Isolation of mutant full-length RHAs. The mutant RHAs containing deletions in the linker region were purified from mammalian cells (HEK 293E), separated by 1D SDS-PAGE, and analyzed by either staining with Coomassie Brilliant Blue R250 (CBR) or probing Western blots with anti-His (WB). Lane M shows a protein size marker with indicated molecular weights in kDa. (B) Diagram of the duplex RNA substrate with one strand 3′-end-labeled with ^32^pCp. (C) EMSA was carried out to examine the *in vitro* binding of purified proteins to [^32^pCp]-labeled synthetic duplex RNA. GST was included as a negative control. Shown is a representative of 3 independent experiments. (D) Helicase activity assay. 10 nM of radioactive duplex RNAs and 150 nM of indicated proteins were incubated at 37°C for indicated time periods in the presence or absence of 1 mM ATP, and then the radioactive RNA strand in single or duplex form was resolved on a 15% native polyacrylamide gel, and visualized using a PhosphorImager. Lane C indicates the migration position of the ssRNA (boiled substrate), while lane N represents the migration position of untreated radioactive duplex RNA. Lanes at time point 0 represent the helicase reactions in the absence of 1 mM ATP. Shown is a representative of 3 independent experiments. (E) Duplex RNA bands in panel D were quantitated using a PhosphorImager, normalized to the results obtained at the time point 0, and presented graphically as percentage. Shown are the mean values ± standard deviations of 3 independent experiments. *, *P* < 0.05 compared with corresponding values obtained with wild-type RHA.

In order to determine whether the linker region of RHA influences either RHA binding to RNA or RHA's helicase activity, we made recombinant plasmids encoding His-tagged mutant RHAs containing deletions of either helix 2, 3, 4 or 5 (Figure 1). We did not analyze RHAs containing deletion of either helix 1 or 6 because of their poor expression in cells. Wild-type and mutant RHAs were purified from 293E cells by affinity chromatography as reported previously [13]. Figure 2A shows the electrophoretic analysis of the purified mutant RHA species. The abilities of wild-type and mutant RHAs to bind to RNA were analyzed by performing EMSA, using [^32^pCp]-3′-end-labeled synthetic duplex RNA. As shown in Figure 2B, this duplex RNA contains a 3′-single-stranded extension which has been shown to be required for initiating helicase activity of RHA [5]. and therefore was also used to assess the enzyme's helicase activity. As shown in Figure 2C, both wild-type and mutant RHAs interact with the [^32^pCp]-labeled duplex RNA with similar efficiency, as indicated by the shift in probe mobility. The specificity of this interaction was evaluated by replacing the RHAs with GST protein, which does not bind to RNA.

We next determined the ability of wild-type or mutant RHAs to unwind the radioactive duplex RNA in the required presence of ATP, using EMSA to resolve duplex from ssRNA. The duplex RNA, labeled on the short RNA strand with ^32^pCp (Figure 2B) was incubated with 150 nM wild-type or mutant RHA at 37°C in the presence of 1 mM ATP. The unwinding reaction was stopped at the indicated time points by proteinase K digestion, and the reaction mixture was then resolved by 1 dimensional polyacrylamide gel electrophoresis (1D-PAGE), using a 15% native polyacrylamide gel. Unwinding activity will release the [^32^pCp]-tagged short RNA strand from the duplex, producing a radioactive band of faster electrophoretic mobility. As shown in Figure 2D, wild-type and mutant RHAs all have the ability to cause a decrease of duplex RNA correlated with an increase of ssRNA, indicating that each mutant RHA still possesses unwinding activity. However, mutant RHAs containing deletion of helices 2 or 3 (but not of helices 4 or 5) show a slower rate of unwinding (Figure 2E), which cannot be explained by a weaker binding of these mutant RHAs to duplex RNA.

Thus, having established *in vitro*, that the mutant RHAs still bind well to RNA, but that deletion of helices 2 or 3 reduces the rate of duplex RNA unwinding, we then tested the biological effect of these mutations upon HIV-1 production in the cell.

### Binding of mutant RHAs to HIV-1 RNA *in vivo*

**Figure 3:**
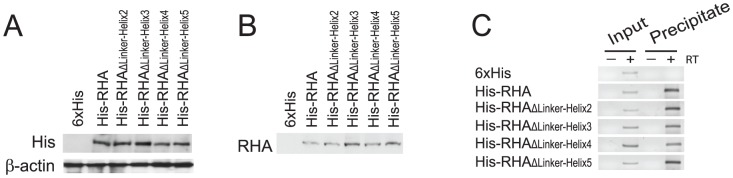
Ability of mutant RHAs to interact with HIV-1 RNA in the cell. 293T cells were transfected with SVC21.BH10 and a plasmid expressing either His-tagged wild-type or mutant RHA, or only the 6×His tag. 24 hours later, cells were cross-linked, lysed, and sonicated. The cell lysates were incubated with Ni-NTA agarose to capture His-tagged protein. RNAs isolated from cell lysates (input) or from nucleoprotein bound to Ni-NTA agarose (precipitate) were subjected to RT-PCR analysis. (A) Western blot of cell lysates was probed with anti-His to detect expression of His-tagged RHA in transfected cells. The expressed 6×His tag peptide alone was not detectable in the Western blot. (B) Western blot of the precipitates was probed with anti-RHA. (C) The input RNA and RNA that was coprecipitated with His-tagged proteins were analyzed by RT-PCR, using primer pair P1-F/R [19] specific to HIV-1 RNA. RT-PCR was performed in the presence (+) or absence (–) of reverse transcriptase.

RHA has been reported to participate in the HIV-1 RNA metabolism at multiple steps [1], [21]. The dsRBDs of RHA are essential for the interaction of RHA with HIV-1 RNA during virus replication [19]. To determine whether the linker region connecting dsRBD2 to the core helicase domain of RHA affects RHA binding to HIV-1 RNA *in vivo*, we performed an RNA-protein coprecipitation assay. 293T cells were cotransfected with SVC21.BH10 containing full-length HIV-1 BH10 proviral DNA and a plasmid expressing His-tagged wild-type or mutant RHA. Cotransfection with SVC21.BH10 and a plasmid expressing only the His tag was also performed as a negative control. 24 hours posttransfection, cells were cross-linked by formaldehyde, lysed, and sonicated. RNA-His-tagged protein complex was precipitated from the cell lysates using Ni-NTA agarose. Total cellular RNA isolated prior to incubation with Ni-NTA agarose (input RNA) and RNA isolated from the His-RHA precipitates (precipitate RNA) was analyzed by RT-PCR using primer pair P1-F/R [19] that is specific for the 5'-UTR of HIV-1 RNA. HIV-1 5′-UTR is the primary region in HIV-1 genomic RNA to which RHA binds during virus replication [19]. Western blots of equal volumes of cell lysates probed with anti-His indicate similar expression of wild-type and mutant RHAs (Figure 3A). Western blots of column eluant probed with anti-RHA indicate comparable amounts of wild-type and mutant RHA in the eluants (Figure 3B). Relative to corresponding input RNA control, RT-PCR readily detected the HIV-1 transcripts containing sequence of HIV-1 5′-UTR that were coprecipitated with either wild-type or mutant RHAs (Figure 3C). The results demonstrate that deletions in the linker region do not prevent the interaction of RHA with HIV-1 RNA in the cells, supporting a similar conclusion reached when studying the *in vitro* binding of mutant RHAs to synthetic duplex RNA (Figure 2).

### Ability of mutant RHAs to stimulate the synthesis of HIV-1 mRNAs

**Figure 4:**
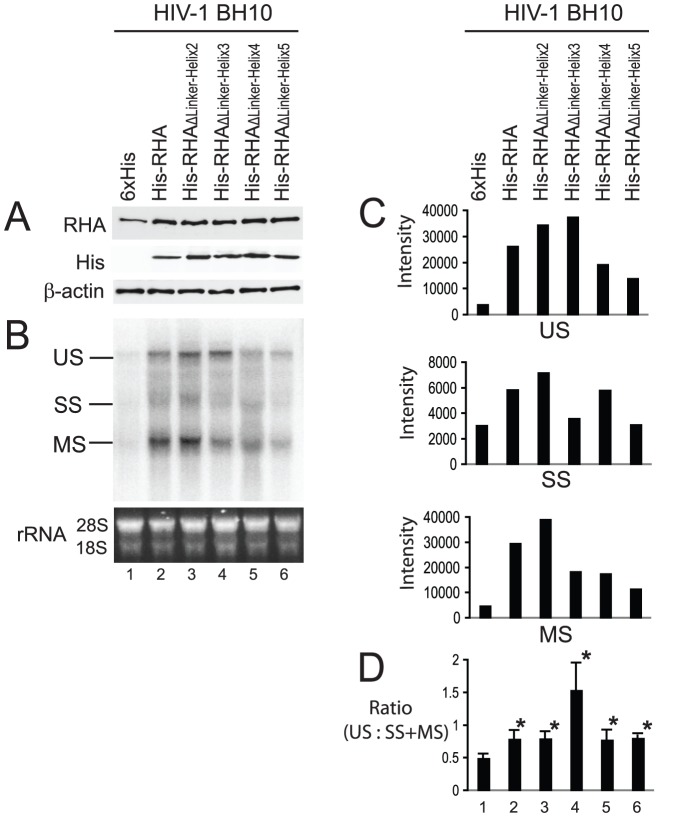
Ability of mutant RHAs to stimulate the synthesis of HIV-1 mRNAs. 293T cells were cotransfected with SVC21.BH10 and either a plasmid expressing His-tagged wild-type or mutant RHA, or only the 6×His tag. 24 hours later, cell lysates and total cellular RNA were prepared and subjected to Western blotting and Northern blotting analysis respectively. (A) Western blots of cell lysates probed with anti-RHA, anti-His, or anti-β-actin. (B) Northern blotting. The total cellular RNA was resolved by electrophoresis on a denaturing 1% agarose gel, and blotted onto GeneScreen Plus membrane. The membrane was probed with the [32P]-labeled DNAs that are complimentary to HIV-1 5'-UTR. Ethidium bromide-stained rRNAs (18S and 28S) are included as an RNA loading control. Unspliced (US) ∼ 9.2 kb, singly spliced (SS) ∼ 4.0 kb, and multiply spliced (MS) ∼ 1.8 kb RNAs are indicated. (C) The intensity of RNA bands in panel B representing US, SS, or MS RNAs was quantitated using a PhosphorImager instrument, and are presented graphically. Shown is a representative of 3 independent experiments. (D) The ratio of US RNA to SS+MS RNA in panel B was determined. Shown are the mean values ± standard deviations of 3 independent experiments. *, *P* < 0.05 compared with values obtained with the 6×His tag alone (lane 1).

Overexpression of RHA in 293T cells has been shown to stimulate the synthesis of HIV-1 mRNA [11]. To elucidate the role of the linker region in this process, 293T cells were cotransfected with SVC21.BH10 and plasmids coding for either wild-type or mutant RHAs. 24 hours later, total cellular RNA was isolated and subjected to Northern blot analysis using [^32^P]-labeled DNA probes that are complementary to the HIV-1 5'-UTR. Cell lysates were also prepared and analyzed by Western blotting using anti-RHA, anti-His, or anti-β-actin to verify the expression of exogenous wild-type or mutant RHAs (Figure 4A). Lane 1 shows the endogenous RHA present. Three different lengths of HIV-1 mRNAs were detected in Northern blot analysis (Figure 4B), representing the multiply spliced (MS, ∼ 1.8 kb), singly spliced (SS, ∼ 4.0 kb), and unspliced (US, ∼ 9.2 kb) RNA size classes. Equivalent cellular RNA loading was confirmed by visualizing 18S and 28S ribosome RNAs after staining with ethidium bromide. The amount of each RNA size class detected by Northern blotting was quantitated using a PhosphorImager, and shown graphically in Figure 4C. The results show that the amounts of HIV-1 mRNA transcripts of all three size classes were increased by coexpression of exogenous wild-type or mutant RHAs. However, overexpression of mutant RHAs containing a deletion of either helix 4 or helix 5 (RHA_ΔLinker-Helix4_ or RHA_ΔLinker-Helix5_) results in less increase in overall mRNA synthesis, indicating that these two helices are required for the stimulation of HIV-1 mRNA synthesis by RHA. Consistent with a previous report [2], we also note that overexpression of RHA favors the accumulation of unspliced viral RNA as the ratio of US RNA to SS + MS RNA was increased upon overexpression of both wild-type and mutant RHAs, especially for mutant RHA with a deletion of helix 3 (Figure 4D, lane 4).

### Ability of mutant RHAs to affect the splicing patterns of HIV-1 mRNAs

**Figure 5:**
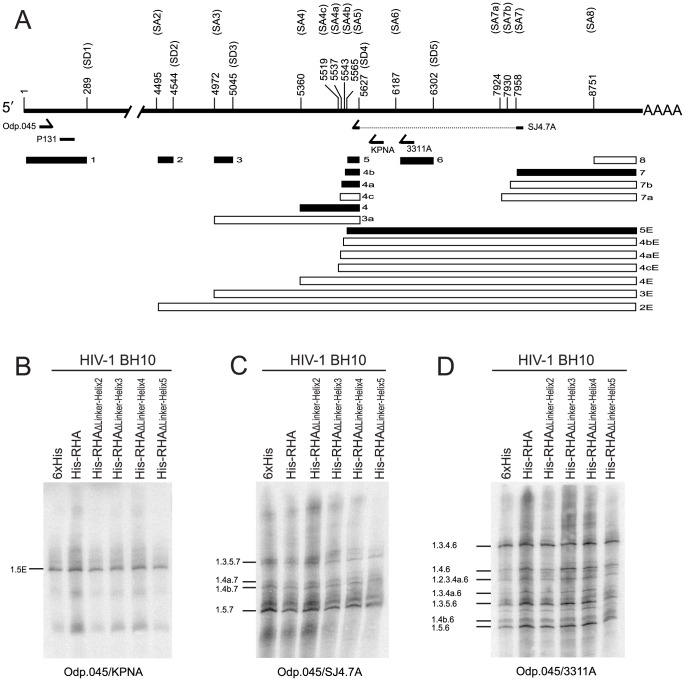
RT-PCR analysis of singly (∼ 4.0 kb) and multiply (∼ 1.8 kb) spliced RNA species. Total cellular RNAs analyzed in Figure 4 were subjected to semiquantitative RT-PCR. 4-16 µl of PCR products were heat-denatured, separated in 6% denaturing polyacrylamide gel, transferred onto GeneScreen Plus membrane, and then probed with [^32^P]-labeled DNA oligonucleotide P131 that can recognize all HIV-1 RNA transcripts. The radioactive signals were visualized using a PhosphorImager. (A) Diagram showing the organization of major splice donor (SD1-5) and acceptor (SA1-8) sites, and the locations of viral exons and oligonucleotide primers on the HIV-1 genomic RNA. Filled boxes represent the exons detected in this study. The viral nucleotide numbers between 1 and 224 correspond to that of human immunodeficiency virus 1 (GenBank accession no. NC_001802). The viral nucleotide numbers between 225 and 9156 correspond to that between 1 and 8932 of human immunodeficiency virus type 1, isolate BH10 genome (GenBank accession no. M15654 K02008 K02009 K02010). (B) Analysis of ∼ 4.0 kb HIV-1 RNA species using primer pair Odp.045/KPNA. (C) Analysis of ∼ 1.8 kb HIV-1 RNA species using primer pair Odp.045/SJ4.7A. (D) Analysis of exon 6D-containing HIV-1 RNA species using primer pair Odp.045/3311A. Shown is a representative of 3 independent experiments.

The primary HIV-1 RNA transcripts undergo complex splicing events during virus replication, producing over 40 different RNA species [22]. Many of these species can be detected in the Northern blot analysis as the US, SS, or MS RNAs shown in Figure 4B, but some are less easily detected. RHA has been implicated in playing a role in splicing because it has been identified as being associated with the spliceosome [10], and because overexpression of RHA increases the ratio of US: SS + MS (Figure 4D, and [2]). Furthermore, we noted that while overexpression of wild-type RHA or mutant RHA_ΔLinker-Helix2_ results in an increase of similar proportions between the different mRNA classes, these proportions are reproducibly diverse for other mutant RHAs (Figure 4C). For example, overexpression of mutant RHA_ΔLinker-Helix4_ results in a better production of SS RNA when compared with mutant RHA_ΔLinker-Helix3_ or RHA_ΔLinker-Helix5_ (Figure 4C). Therefore, we have determined whether overexpression of wild-type or mutant RHAs will alter the splicing pattern that produces mRNAs in the SS and MS classes by performing splicing analysis as described in detail in Material and Methods. It has been shown that the SS and MS classes of transcripts consist of at least 14 differently spliced members for each class [22]. Figure 5A shows the locations of the viral exons and the primers used in RT-PCR to specifically amplify the different mRNA size classes. Three sets of primers were used to amplify the three different classes of viral mRNAs, i.e. the SS mRNA (Odp.045/KPNA, Figure 5B), MS mRNA (Odp.045/SJ4.7A, Figure 5C), or MS mRNAs containing exon 6D (Odp.045/3311A, Figure 5D). The results show that the splicing pattern for each group of RNA species as detected using a respective primer pair is similar between different RNA samples, indicating that overexpression of either wild-type or mutant RHA does not apparently affect the usage of a specific splice donor or acceptor site within each group of mRNAs. Additionally, as shown in Figure 5D, a number of exon 6D-containing spliced viral RNA transcripts were also detected by RT-PCR in HIV-1 BH10-producing 293T cells. Sequencing analysis confirms that some of them may encode chimeric protein Tev (Tat-Env-Rev fusion protein) [23], [24]. The partial sequence of some of these transcripts isolated in this study was deposited in GenBank (accession no. KC493108, KC493109, KC493110, KC493111, KC493112, or KC493113).

### Ability of mutant RHAs to be incorporated into virion, and to promote the annealing of tRNA^Lys3^ to viral RNA

**Figure 6:**
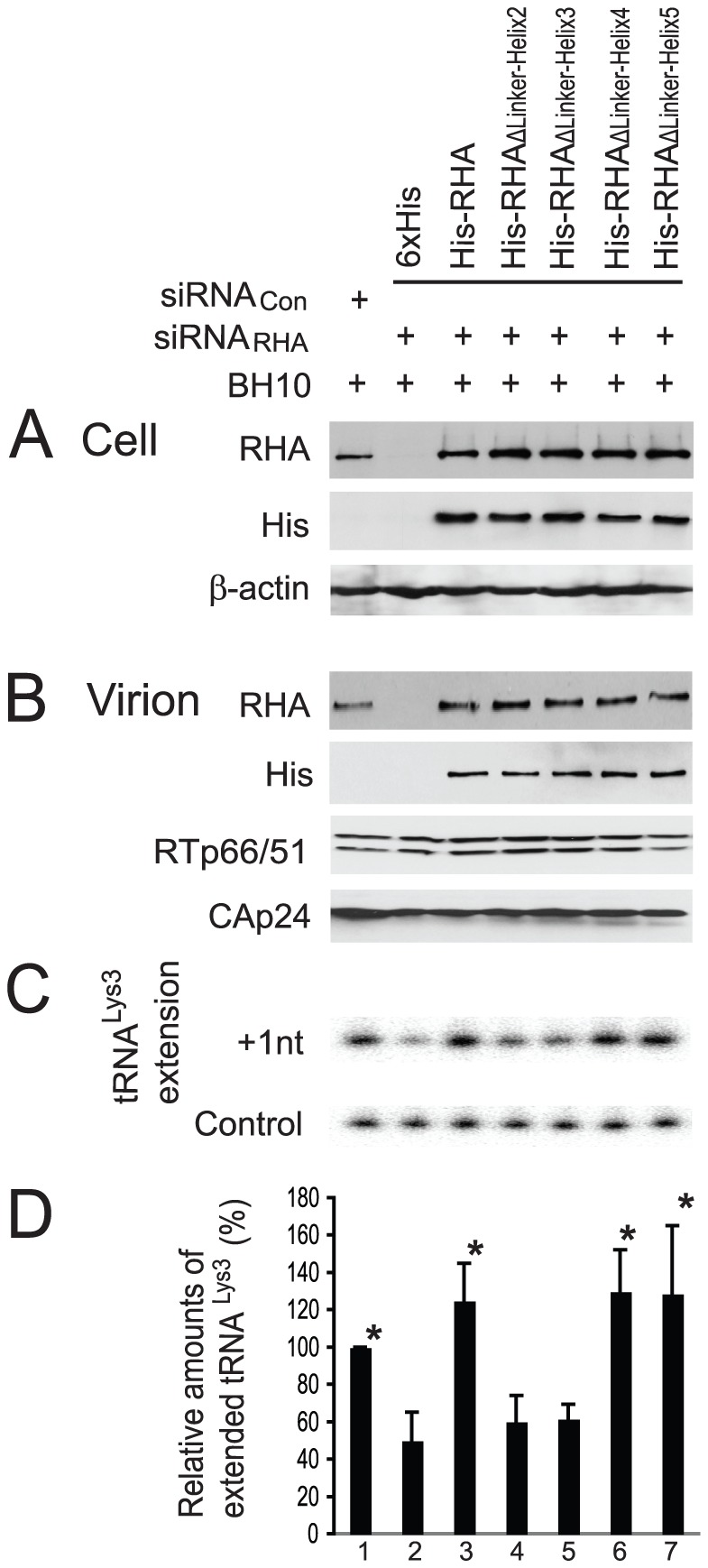
Ability of mutant RHAs to promote the annealing of tRNA^Lys3^ to viral RNA. 293T cells were first treated with siRNA_Con_ or siRNA_RHA_, and 16 hours later, were cotransfected with SVC21.BH10 and a plasmid expressing either 6×His tag, or His-tagged wild-type or mutant RHAs. 48 hours later, extracellular viruses were purified and cells were lysed. (A) Western blots of cell lysates probed with antibodies to RHA, His tag, or β-actin. (B) Western blots of viral lysates, containing equal amount of CAp24, probed with antibodies to RHA, His tag, CAp24, or RTp66/p51. (C) One nucleotide extension assay (+1 nt extension). Total viral RNA was isolated from purified HIV-1 particles, and tRNA^Lys3^ annealed to viral RNA *in vivo* was extended by 1 nt ([^32^P]-dCTP), using HIV-1 reverse transcriptase. The extended tRNA^Lys3^ products are resolved by denaturing 1D PAGE, and visualized using a PhosphorImager. The control gel represents the +1 nt extension of a DNA primer annealed *in vitro* to viral RNA downstream of the tRNA^Lys3^ binding site, and is used to show that approximately equal amounts of viral RNA were used in each extension reaction. (D) The values of the +1 nt extended tRNA^Lys3^ products were quantitated using a PhosphorImager, normalized to the values obtained with virions produced from siRNA_Con_-treated cells (lane 1), and are presented graphically as a percentage. Shown are the mean values ± standard deviations of 3 independent experiments. *, *P* < 0.05 compared with values obtained with virions produced from cells transfected with a plasmid expressing only His tag (lane 2).

Depletion of endogenous RHA by siRNA_RHA_ reduces the annealing of tRNA^Lys3^ to viral RNA, and this defect in tRNA^Lys3^ annealing could be rescued by expression of exogenous RHA whose mRNA lacks the siRNA_RHA_ target sequences [13], [14]. Since the mRNAs encoding the mutant RHAs in this report also lack siRNA_RHA_ target sequences, the one nucleotide extension assay of annealed tRNA^Lys3^ was carried out to examine the ability of each mutant RHA to rescue reduced tRNA^Lys3^ annealing by siRNA_RHA_ (Figure 6). Initially, the endogenous RHA in 293T cells was depleted by siRNA_RHA_ treatment. 16 hours later, cells were cotransfected with SVC21.BH10 and RHA constructs expressing either His-tagged wild-type or mutant RHAs. 48 hours posttransfection, the extracellular viral particles were purified, and cell and viral lysates were analyzed by Western blotting using anti-RHA, anti-His, anti-β-actin, anti-RTp66/p51, or anti-CAp24. The results verified that the siRNA_RHA_ depleted the endogenous RHA in the cell (Figure 6A, lane 2), but not the exogenous wild-type or mutant RHAs (Figure 6A, lanes 3–7). Western blots of viral lysates probed with anti-RHA or anti-His revealed that both wild-type and mutant exogenous RHAs were detected in the virus particles (Figure 6B), indicating that the deletions in the linker region do not affect the ability of RHA to be incorporated into virus particles.

Next, the one nucleotide extension assay was carried out using total viral RNA isolated from purified HIV-1 particles as the source of primer tRNA^Lys3^ annealed *in vivo* to viral genomic RNA. The incorporation of the first dNTP, [^32^P]-dCTP, by reverse transcription *in vitro,* is detected by 1D PAGE. In this assay, equal amounts of viral genomic RNA used in the reactions were first quantitated by dot blot hybridization, and further validated by a one nucleotide extension of an annealed DNA primer complementary to viral RNA sequences downstream of the tRNA^Lys3^ binding site (Figure 6C, control panel). The 1D PAGE resolution of tRNA^Lys3^ extensions shown in Figure 6C (+1 nt panel) was quantitated using a PhosphorImager, and normalized to results obtained with siRNA_Con_, and the results are shown graphically in Figure 6D. The normal annealing (lane 1) was reduced by the presence of siRNA_RHA_ (lane 2), and rescued by either exogenous wild-type RHA (lane 3), or mutant RHA_ΔLinker-Helix4_ or RHA_ΔLinker-Helix5_ (lanes 6 or 7). By contrast, little rescue of annealing was obtained by expression of the mutant RHA_ΔLinker-Helix2_ or RHA_ΔLinker-Helix3_ that contains a deletion of either helix 2 or 3 (lanes 4 or 5). These results indicate that helices 2 and 3 are required for the promotion of tRNA^Lys3^ annealing to viral RNA by RHA, but none of the helices in the linker region analyzed in this report are required for the RHA packaging into HIV-1 particles.

## Discussion

In this report, as summarized in Table 1, we found that the linker region that connects dsRBD2 with the core helicase domain of RHA possesses diverse roles in HIV-1 RNA metabolism. Analysis of mutant RHAs containing individual deletions of 4 of the 6 helices in the linker region indicates that this region is not required for the binding of RHA to substrate RNA *in vitro* (Figure 2C) and *in vivo* (Figure 3C). While *in vitro* analysis showed that none of the deletions in the linker region eliminated the helicase activity of RHA, deletion of helices 2 or 3 reduced the rate of RNA unwinding (Figure 2D, E). It is not clear how these deletions in the linker region reduced the helicase activity of RHA. The core helicase domain is usually responsible for the RNA unwinding activity of DExH RNA helicases to which RHA belongs [25]. This domain in RHA is also well conserved in amino acid sequence as well as in structure [26]. However, RHA is distinguished by two dsRBDs at the amino terminus in the group of DExH RNA helicases. Thus, it is likely that the linker region connecting dsRBDs to the core helicase domain exerts regulatory role in helicase activity of RHA, instead of directly participating in RNA strand separation.

**Table 1:** Effect of mutations in the linker region of RHA upon helicase activity *in vitro* and upon multiple steps in HIV-1 production in the cells*^a^*.

Parameters	Helicase activity	Interaction with HIV-1 RNA in the cells	Incorporation into virion	Increase in ratio of unspliced HIV-1 RNA to spliced RNA	Stimulation of HIV-1 RNA synthesis	Promotion of tRNA^Lys3^ annealing
RHA wt	+	+	+	+	+	+
ΔHelix 2	±	+	+	+	+	−
ΔHelix 3	±	+	+	+	+	−
ΔHelix 4	+	+	+	+	±	+
ΔHelix 5	+	+	+	+	±	+

a± ±, Partial reduction.

We have found that RHA linker region is implicated in different ways for the multiple processes of HIV-1 RNA metabolism that are regulated by sequences in the HIV-1 5′-UTR, the region to which RHA most strongly binds [19]. In particular, the observation that RHA promotes both viral transcription and tRNA^Lys3^ annealing is reminiscent of the HIV Tat protein. The Tat protein, which is a well-known transcriptional trans-activator and is essential for viral transcription [27], [28], also has an RNA-annealing activity [29] and can promote the placement of tRNA^Lys3^ onto viral RNA in an *in vitro* analysis [30]. Tat stimulates HIV transcription by specifically binding to TAR and U3 elements [31]. However, the mechanism(s) used by RHA to stimulate HIV-1 transcription may be complex, because it has been shown that both RHA helicase activity and an RHA bridge between RNA polymerase II (RNAP II) and the CREB binding protein (CBP) are required for the up-regulation of transcription of many cellular genes [11]. In HIV-1 replication, the CBP has been implicated in the activation of HIV-1 RNA transcription [32], [33]. By analysis of cellular genes, it has been shown that RHA stimulates CREB-dependent transcription by facilitating the recruitment of RNAP II to CBP via a minimal transactivation domain (MTAD) in RHA [34], [35]. The MTAD contains about 50 amino acid residues in length between amino acid positions 331 and 380 and has transcriptional activity in both yeast and mammalian cells, and interacts with RNAP II. The fact that mutant RHA containing deletions of either helix 4 or helix 5 possesses full helicase activity similar to wild-type RHA (Figure 2), but is nevertheless less stimulatory to HIV-1 RNA synthesis than wild-type RHA (Figure 4) may be related to the fact that Helix 4 is located within the MTAD and helix 5 overlaps the C-terminal boundary of MTAD. By contrast, it is only the mutant RHA containing deletion of either helix 2 or helix 3, that is not able to efficiently rescue the reduction in the annealing of tRNA^Lys3^ to viral RNA caused by depletion of endogenous RHA by siRNA_RHA_ (Figure 6). These two mutant RHAs have a reduced helicase activity *in vitro* (Figure 2). Since helicase activity is required for RHA to promote the annealing of tRNA^Lys3^ to viral RNA both *in vitro* and *in vivo* [13], the observation in this study indicates that even the partial reduction in helicase activity observed *in vitro* can significantly affect the *in vivo* role of RHA in the annealing of tRNA^Lys3^ to viral RNA.

HIV-1 replication produces many differently spliced RNA species that have been classified as US (∼ 9.2 kb), SS (∼ 4.0 kb), or MS (∼ 1.8 kb) RNAs [22]. The US RNA serves as the translation template to produce Gag and GagPol proteins, and it is also packaged into the HIV-1 particles as genomic RNA. SS RNA encodes viral Env, Vpu, Vpr, or Vif protein, whereas MS RNA encodes Tat, Rev, and Nef proteins. Thus, the balanced and coordinated alternative splicing of HIV-1 RNA is critical for virus replication [22], [36]. In eukaryotic cells, a number of RNA helicases have been found to play essential roles in regulation of both constitutive and alternative RNA splicing [37]. For example, *Saccharomyces cerevisiae* Prp22p, a DExH box RNA helicase, is required for both the second transesterification step and the release of mature mRNA from the spliceosome [38]. DEAD-box RNA helicase p72 affects the splicing of alternative exons containing AC-rich exon enhancer elements [39]. Particularly, RNA helicase p68 was recently reported interacting with a stem-loop structure at the splice site of tau exon 10 and works as an activator of tau exon 10 splicing in tauopathy [40]. RHA has also been identified to be associated with the spliceosome [10] and implicated in the release of US HIV-1 RNA from the spliceosome [2]. However, neither wild-type nor mutant RHA containing a deletion in the linker region affects the usage of a specific splice donor or acceptor site within SS or MS HIV-1 mRNAs (Figure 5). These observations show that RHA may not widely affect the usage of splice donor and acceptor sites throughout the viral genome, suggesting that the effects of overexpression of RHA upon the increased accumulation of US HIV-1 RNA are mainly due to a specific binding of RHA to the 5'-UTR of HIV-1 RNA [19], leading to a locally suppressed usage of the first major splice donor SD1 sequence within this region.

In conclusion, the differential responses to the mutations in the linker region of RHA in this report reveal that RHA participates in multiple steps of HIV-1 RNA metabolism by multiple distinct mechanisms.

## Materials and Methods

### Nucleotide sequences

The partial sequence of HIV-1 RNA transcripts containing exon 6D was deposited in GenBank (accession no. KC493108, KC493109, KC493110, KC493111, KC493112, or KC493113).

### Cell culture

293T, a human embryonic kidney cell line, was maintained in Dulbecco's modified Eagle's medium (DMEM, Invitrogen) supplemented with L-glutamine (Gibco), penicillin-streptomycin (Gibco), and 10% fetal bovine serum. HEK 293E cells are a stably transfected 293 cell line [41] and were used to purify the enzymatically active wild-type or mutant RHA. This cell line expresses the Epstein-Barr virus nuclear antigen 1 (EBNA1) and supports the amplification of plasmid backbone pTT5-SH5 containing the replication origin region (OriP) of Epstein-Barr virus.

### Plasmids

SVC21.BH10 is a simian virus 40-based vector that contains full-length wild-type HIV-1 BH10 proviral DNA [42]. Other recombinant plasmids were generated by fusion PCR and verified by performing restriction mapping and DNA sequencing. The gel-purified PCR products were digested by NotI and SapI, and then cloned into pTT5-SH5-RHA [13] that contains full-length human RHA coding sequence under control of a CMV promoter. This plasmid was also used as a template in PCR amplification. The recombinant plasmids pRHA-Δlinker-Helix2, pRHA-Δlinker-Helix3, pRHA-Δlinker-Helix4, or pRHA-Δlinker-Helix5 encode mutant RHAs containing deletion of predicted helices 2, 3, 4, or 5 in the linker region, respectively, and were generated by fusing PCR product of primers RHA-F1(4) and Linker-H2-R, Linker-H3-R, Linker-H4-R, or Linker-H5-R to that of primers RHA-SapI-R(1400) and Linker-H2-F, Linker-H3-F, Linker-H4-F, or Linker-H5-F, respectively. Primer RHA-F1(4) has been described [19]. Other primers are listed in Table 2.

**Table 2:** Primers used in fusion PCR to construct recombinant plasmids.

Primer	Sequences (5′―3′)
RHA-SapI-R(1400)	TTTCCAGGCTCTTCTCCTCT
Linker-H2-F	CCGCCTGAAGATCCTTCTAACATTGGCAAATTGGCT
Linker-H2-R	AGAAGGATCTTCAGGCGG
Linker-H3-F	GCTCAGTTCGAACCATCTAACCAAGTGGGTGTGGTT
Linker-H3-R	AGATGGTTCGAACTGAGC
Linker-H4-F	CCTCTGGCTTTTGCTACTCAGGATCATGATTTGCAA
Linker-H4-R	AGTAGCAAAAGCCAGAGG
Linker-H5-F	TACCAGTTGGAACAGGATGAGTTACTGCCTGTGAAG
Linker-H5-R	ATCCTGTTCCAACTGGTA

### Expression and purification of proteins

The N-terminally 6×His-tagged mutant RHAs containing deletions in the linker region were expressed and isolated as described [13] from HEK 293E cells [43]. Briefly, cells were transfected with indicated plasmids using 25 kDa linear Polyethylenimine (PEI, pH7.0, Polysciences Inc.), collected 48 hours later, washed with ice-cold phosphate-buffered saline, and lysed in lysis buffer [50 mM NaH_2_PO_4_, pH 7.4, 300 mM NaCl, 10 mM imidazole, 0.5% Triton-X 100, 10% glycerol, and protease inhibitor cocktail tablets (Roche)]. The cell lysates were cleared by centrifugation and then incubated with Ni-nitrilotriacetic acid (NTA) agarose (Qiagen) at 4°C for 2 hours to capture His-tagged proteins. After extensive washing, recombinant proteins were eluted by 250 mM imidazole solution (pH 7.4). N-terminally 6×His-tagged full-length RHA without deletion mutation was referred to as wild-type (WT) in this study, and has been described previously [13]. Glutathione S-transferase (GST) was isolated from HEK 293E cells as described previously [19]. The purified proteins were dialyzed in dialysis buffer [20 mM Tris-HCl, pH 7.5, 150 mM NaCl, 20 mM KCl, 2 mM MgCl_2_, 2 mM dithiothreitol, 10% glycerol] and then stored at –80°C. The purity and the identity of purified protein were determined by Coomassie Brilliant Blue R250 staining and Western blot analysis using anti-His, respectively. Protein concentration was determined using the Bio-Rad protein assay reagent.

### siRNA and Western blot analysis

Small interfering RNA oligonucleotides (siRNA) were employed to knockdown the endogenous RHA in 293T cells as previously described [13]. Viral or cell lysates were analyzed by Western blotting using appropriate primary antibodies including rabbit anti-HIV RT, mouse anti-CAp24 (NIH AIDS Research and Reference Reagent Program), β-actin mAb (Sigma), RHA mAb (M01, Abnova Inc.), and poly-histidine mAb (Sigma).

### RNA-protein coprecipitation assay

The RNA-protein coprecipitation assay was performed to examine the RNA-protein interaction *in vivo* as described [19]. 293T cells were transfected with SVC21.BH10 and a plasmid expressing 6×His-tagged wild-type or mutant RHA. 24 hours later, the cells were cross-linked in 1% formaldehyde (Bioshop), lysed in lysis buffer containing 50 mM Tris-HCl, pH 7.5, 150 mM KCl, 1% NP-40, 0.1% SDS, 0.5% sodium deoxycholate, 50 mM NaF, 1000 U/ml SUPERase-in (Ambion), and protease inhibitor cocktail tablets (Roche), sonicated, and then centrifuged. The cleared supernatants were incubated with salmon sperm DNA and mammalian RNA-saturated Ni-NTA agarose to capture His-tagged protein. Ni-NTA agarose was then collected and extensively washed. The recombinant proteins were eluted with 250 mM imidazole, pH7.4, reverse cross-linked, and then extracted with TRIzol reagent (Life technology) to isolate RNA. Purified RNA (2%) before incubation with Ni-NTA agarose was used as an input control. The RNA sample was treated with DNase and then subjected to semiquantitative reverse transcriptase (RT)-PCR analysis using the Superscript II RT kit (Invitrogen), Taq DNA polymerase (Bio basic Canada, Inc.), and primer pair P1-F/R specific for HIV-1 5′-untranslated region (UTR) [19]. The PCR amplification was started with an initial denaturation at 94°C for 5 min, followed by 25 cycles at 94°C for 30 s, 52°C for 30 s, and 72°C for 20 s, and finalized with incubation at 72°C for 5 min. PCR products were analyzed in 1.5% agarose gel containing ethidium bromide.

### One nucleotide extension assay

The tRNA^Lys3^-viral RNA template was extracted from purified HIV-1 particles as described [44]. The ability of tRNA^Lys3^ annealed *in vivo* to viral RNA to be extended by one nucleotide (dCTP) by reverse transcription was assayed using a 20 µl reaction volume containing 50 mM Tris-HCl, pH 7.8, 100 mM KCl, 10 mM MgCl_2_, 10 mM dithiothreitol, 0.16 µM [α-^32^P]-dCTP, 50 ng of HIV-1 RT, and RNase inhibitor (Ambion). The reaction was incubated for 15 min at 37°C. The RNAs in the reaction were precipitated with 2-propanol, separated in a denaturing 6% polyacrylamide gel. The relative amounts of extended radioactive tRNA^Lys3^ were determined using a PhosphorImager instrument. The relative amount of viral RNA used in the reactions was also determined by measuring the ability of a DNA oligonucleotide (5′-TCTAATTCTCCCCCGCTTAATACTGACGCT-3′) annealed at room temperature to the viral RNA sequence downstream of the tRNA^Lys3^ binding site to prime a one base ([α-^32^P]-dCTP) extension.

### Preparation of RNA substrates

A duplex RNA was used to measure the helicase activity of wild-type or mutant RHA purified from mammalian cells. One 34-nt sense RNA oligonucleotide has been described [13]. The antisense RNA oligonucleotide was generated by *in vitro* transcription using T7 RNA polymerase (T7 MEGAscript kit, Ambion). The DNA template for *in vitro* transcription was amplified by PCR from a luciferase open reading frame (ORF) in pGL3-basic (Promega) using primer pair LucMb-F (5'-CGCTAATACGACTCACTATAGGGAGACAGTGCTGCAATGATACC-3', forward, T7 promoter region is underlined)/LucMb-R (5'-TTTATTGCTGATAAATCTGGGGAGAGAGCCGGTGAGCGTG-3', reverse). The sense RNA strand was labeled with [5'-^32^P]cytidine 3',5'-bis(phosphate) (^32^pCp) using T4 RNA ligase (Fermentas), and then annealed to cold complementary antisense RNA strand to produce radioactive duplex RNA (diagramed in Figure 2B). The radioactive duplex RNA used in electrophoretic helicase assays was first purified by electrophoresis on a 15% native polyacrylamide gel in 0.5×Tris-borate-EDTA (TBE) followed by extraction using RNA polygel extraction kit (Biomiga).

### Electrophoretic mobility shift assay (EMSA) and electrophoretic helicase assay

EMSA was performed to examine the ability of wild-type or mutant RHA to bind dsRNA. 100 nM [^32^pCp]-labeled duplex RNAs were incubated with varied amounts of protein (2 nM, 4 nM, 8 nM, and 20 nM) at room temperature for 30 min in 20 µl of binding buffer (20 mM Tris, pH 7.4, 5% glycerol, 50 mM KCl, 150 mM NaCl, 10 mM MgCl_2_, and 2 mM dithiothreitol). The RNA-protein mixture was then separated in a 5% native polyacrylamide gel in 0.5×TBE. Helicase activity of RHA was measured by performing electrophoretic helicase assay as described [13] in a 20 µl reaction containing 10 mM Tris-HCl, pH 8.0, 50 mM KCl, 2 mM MgCl_2_, 2 mM dithiothreitol, 2 units of RNasin, 2.5 mM NaH_2_PO_4_, 15 mM NaCl, 10 nM gel-purified duplex RNA, and, where indicated, 1 mM ATP, and 150 nM purified proteins. The reaction was incubated at 37°C for indicated time periods and then stopped by adding 5 µl of stop buffer (2% SDS, 10 mM CaCl_2_, 250 µg/ml proteinase K, 40% glycerol, 0.1% bromphenol blue, and 0.1% xylene cyanol). 10 µl of aliquots for each reaction were separated in a 15% native polyacrylamide gel in 0.5×TBE. Radioactive RNAs were visualized and quantitated using a PhosphorImager instrument.

### RNA isolation and Northern analysis

RNA isolation and Northern analysis were performed as described previously [45]. Briefly, 293T cells were cotransfected with SVC21.BH10 and a plasmid expressing His-tagged wild-type or mutant RHA. 24 hours later, total cellular RNA was isolated using TRIzol reagent, and 15 ug of RNAs per lane were separated in denaturing 1% agarose-2.2 M formaldehyde gel, transferred onto GeneScreen Plus hybridization transfer membranes (Perkin-Elmer), immobilized by UV light cross-linking, and probed with [^32^P]-labeled DNA probes. The radioactive probes were prepared using random primer DNA labeling system (Invitrogen) and DNA template containing sequence of HIV-1 5′-UTR. These probes can recognize all the HIV-1 transcripts produced during virus replication.

### Splicing analysis

HIV-1 RNA Splicing was analyzed by performing RT-PCR as in principle described previously [22] with modifications. Five primers specific to HIV-1 were used in this assay. Primers Odp.045 [22] and P131 (5'-TAACTAGAGATCCCTCAGAC-3') are located in the 5′-UTR of HIV-1 upstream of the major splice donor SD1 at nt 289 relative to the +1 of the mRNA start site. Primers KPNA, 3311A, and SJ4.7A were designed by Neumann et al [46]. Primer pair Odp.045/SJ4.7A amplifies most of the multiply spliced HIV-1 mRNAs (∼ 1.8 kb). Primer pair Odp.045/KPNA amplifies all the singly spliced HIV-1 mRNAs (∼ 4.0 kb), whereas primer pair Odp.045/3311A amplifies HIV-1 mRNAs containing exon 6D. The cDNAs were initially synthesized from 1 ug of total RNAs using SuperScript reverse transcriptase (Invitrogen) and a 18-mer oligo-dT primer. PCR amplification using Taq DNA polymerase was started by denaturing at 94°C for 4 min, followed by 20 cycles of 94°C for 30 sec, 58°C for 40 sec, and 72°C for 2 min, and finalized by an extension at 72°C for 5 min. PCR products were then denatured at 94°C for 5 min, separated by electrophoresis on a 6% denaturing polyacrylamide gel, transferred onto GeneScreen Plus hybridization transfer membranes, immobilized by UV light cross-linking, and probed with [γ^32^P]-labeled oligonucleotide P131. The PCR products were also purified, cloned into pJET1.2 using CloneJET PCR cloning kit (Fermentas), and then sequenced to determine their identities.

### Statistical analysis

Statistical analysis was performed by Student's *t* test. *P* value < 0.05 was considered significant and was indicated with asterisks.
